# Fecal Virome of Southeastern Maned Sloth (*Bradypus crinitus)* (Pilosa: Bradypodidae)

**DOI:** 10.1590/1678-4685-GMB-2024-0183

**Published:** 2025-05-09

**Authors:** Amanda Coimbra, Mirela D’arc, Filipe Romero Rebello Moreira, Matheus Augusto Calvano Cosentino, Francine Bittencourt Schiffler, Thamiris dos Santos Miranda, Ricardo Mouta, Déa Luiza Girardi, Victor Wanderkoke, Gabriel Medeiros, Talitha Mayumi Francisco, Flávio Landim Soffiati, Suelen Sanches Ferreira, Carlos Ramon Ruiz-Miranda, Marcelo Alves Soares, André Felipe dos Santos

**Affiliations:** 1 Universidade Federal do Rio de Janeiro (UFRJ), Laboratório de Diversidade e Doenças Virais, Rio de Janeiro, RJ, Brazil.; 2 Universidade Estadual do Norte Fluminense Darcy Ribeiro (UENF), Laboratório de Ciências Ambientais, Campos dos Goytacazes, RJ, Brazil.; 3 Associação Mico-Leão-Dourado, Silva Jardim, Rio de Janeiro, RJ, Brazil.; 4 Instituto Nacional de Câncer, Programa de Oncovirologia, Rio de Janeiro, RJ, Brazil.

**Keywords:** Virosphere, Papillomaviridae, Retroviridae, gut microbiome, Southeastern Maned Sloth

## Abstract

We report a viral metagenomic analysis of fecal samples from *Bradypus crinitus* (Pilosa: Bradypodidae), a recently described sloth species that occurs in the Atlantic Forest of Espírito Santo and Rio de Janeiro states, Southeast Brazil. Through Illumina sequencing, we generated a total of 2,065,344 raw reads, of which 945,386 reads (45.77%) passed the quality and size filter. The highest proportion of them was assigned to Eukarya, followed by Bacteria and only a small proportion to Virus. However, we identified 24 viral families using distinct taxonomic assignment tools, including phages and vertebrate viruses, such as retroviruses and papillomaviruses. Also, we identified four bacterial genus already associated with disease in sloths. Our study sheds light on the microbiome of a previously unexplored species, further contributing to the comprehension of metagenomic global diversity.

The maned sloth is endemic to the Brazilian Atlantic Rain forest and has been categorized as vulnerable by the International Union for Conservation of Nature (Chiarello and Moraes-Barros, 2013). A recent taxonomic revision divided the group into two species: northeastern maned sloth (*Bradypus torquatus*), occurring in the states of Bahia and Sergipe; and southeastern maned sloth (*Bradypus crinitus*), in the states of Espírito Santo and Rio de Janeiro, respectively (Miranda et al., 2023). Serology studies elucidated that northeastern collared sloths are well known reservoirs of several arboviruses genera: *Orthobunyavirus*, as Utinga virus (UTIV) (Seymour, 1985) and Caraparu virus (CARV) (Catenacci et al., 2018); *Flavivirus*, as Dengue virus (DENV 1 to 4) (Catenacci *et al.*, 2018), Rocio virus (ROCV) (Catenacci *et al.*, 2018), Bussuquara virus (BSQV) (Catenacci *et al.*, 2018), Saint Louis encephalitis virus (SLEV) (Seymour, 1985), Ilheus virus (ILHV) (Seymour, 1985) and Yellow fever virus (YFV) (Catenacci *et al.*, 2018); and also, *Alphavirus*, as Eastern equine encephalitis virus (EEEV) (Catenacci *et al.*, 2018). However, apart from arbovirus, little is known about the natural viral diversity of maned sloths. Recent advances in high-throughput sequencing (HTS) technologies have allowed comprehensive access to the virosphere, helping to fill gaps in the diversity of eukaryotic viruses (Zhang et al., 2018; Harvey and Holmes, 2022). There are only a few studies that used this technique to assess the enteric virome of sloths (Dill-McFarland et al., 2016; Rojas-Gätjens et al., 2022). Here, we describe the viral diversity obtained by HTS from fecal samples from southeastern maned sloths, collected in the Atlantic Forest of Rio de Janeiro.

The study was carried out between November 2018 and July 2019 in the localities of *Igarapé* and *Dois irmãos* Farms, in the state of Rio de Janeiro (Table 1). Seven apparently healthy adult and subadult southeastern ground sloths were located by active search and monitored directly from the treetops. Fecal samples were collected opportunistically directly from the ground and transferred to 50 mL Falcon tubes, where they were homogenized with an approximate 1:1 volumetric ratio of RNAlater™ (Thermo Fisher Scientific, Walham, USA). The animals were monitored and the samples handled under the approval and legal consent of the Brazilian Federal Authority (numbers 67274-8 and 64635-5). Samples were kept at room temperature and shipped to the *Laboratório de Diversidade e Doenças Virais* (LDDV) at *Universidade Federal do Rio de Janeiro* (UFRJ), Rio de Janeiro, Brazil, to be stored at -80 ºC. For the virome protocol, the samples were vigorously vortexed until complete homogenization. Then, approximately 1 mL of sample was transferred to the extraction bead tube (MP Biomedicals, CA, USA) to break up the debris. The supernatant was collected from each sample and an equimolar volume was pooled in one single tube to maximize sequencing efficiency. The next step was Illumina library construction, following the general CDC protocol methodology (Kohl et al., 2015) with previously described modifications (Cosentino et al., 2022). HTS was conducted on an Illumina MiSeq platform using the MiSeq V2 cartridge with pair-ended 2x151 cycles. Sequencing data was processed with a custom pipeline, also previously described (Cosentino *et al.*, 2022). To avoid false positive results due to sequencing index-hoping (incorrect assignment of reads to a given sample) (Farouni et al., 2020), we considered as invalid the identification of a viral family when it was detected by a number of reads that was less than 1% of the highest count identified for the same family among all other libraries sequenced in the same run. 

**Table 1 -:** General information of collected specimens.

ID	Date	Location	GPS*	Age	Sex
BT01	Nov/2018	Igarapé, Silva Jardim, RJ	-22.50713, -42.30954	Subadult	F
BT03	July/2019	Dois irmãos, Silva Jardim, RJ	-22.38538, -42.27638	Subadult	M
BT05	June/2019	Igarapé, Silva Jardim, RJ	-22.42530, -42.02737	Subadult	M
BT06	June/2019	Dois irmãos, Silva Jardim, RJ	-22.50713, -42.30954	Adult	F
BT07	July/2019	Dois irmãos, Silva Jardim, RJ	-22.38538, -42.27638	Adult	F
BT08	July/2019	Dois irmãos, Silva Jardim, RJ	-22.38538, -42.27638	Adult	F
BT09	July/2019	Igarapé, Silva Jardim, RJ	-22.38538, -42.27638	Subadult	F

*Global Positioning System in Decimal Degrees format.

The pooled samples generated a total of 2,065,344 raw reads, of which 945,386 reads (45.77%) passed the quality and size filter. Taxonomic assignments were performed with both Kraken2 (Wood and Salzberg, 2014) (standard database) and Diamond v.2.0.14 (Buchfink et al., 2015) (NCBI nr database). Using Kraken2, a total of 368,564 reads (38.98%) were classified, being the highest proportion assigned to Eukarya (253,144 reads; 68.7%), followed by Bacteria (113,958 reads; 30.9%) and only a small proportion to Virus (318 reads; 0.09%) (Table S1). Among the viral reads, 24 families were identified (Figure 1; Table S2). Diamond classified 98,694 reads (10.44%), the majority were bacterial (54,910 reads; 55.6%), followed by eukaryotes (42,975 reads; 43.5%) and, similar to Kraken2, a small proportion of virus (294 reads; 0.3%) (Table S1). Preliminary analyses of the bacteriome identified bacterial reads already recognized in sloths such as *Bordetella*, *Citrobacter*, *Escherichia*, *Salmonella*, *Coxiella*, *Anaplasma* and *Ehrlichia* (Table S3), of which the first three and some strains of the fourth were able to cause disease in these animals (Smith and Ruple, 2021).

**Figure 1 -:**
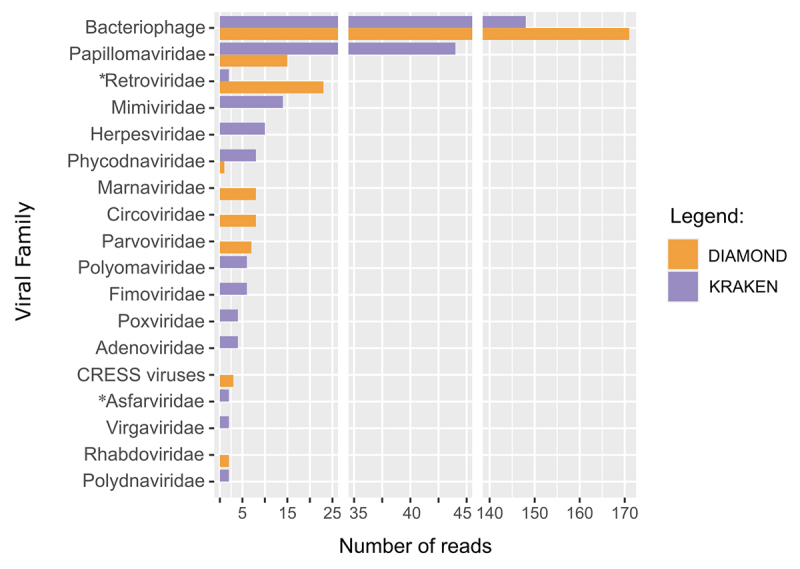
Viral families identified in sloth fecal samples.

*Family with one contig assembled.

A total of 15 viral families were identified by Diamond, and nine (60%) were in agreement with Kraken2. Given the size and abundance of the obtained reads, we considered only the viral families confirmed by both taxonomic identification tools to be reliable. Among the nine viral reads validated by both tools, the bacteriophages were the most abundant (Autographiviridae, Microviridae, Myoviridae, Podoviridae, Salasmaviridae, Schitoviridae and Siphoviridae). This topic will be addressed in a future study to further explore their relationship with the associated bacteriome. The vertebrate viruses, including sequences from Papillomaviridae and Retroviridae, are commonly linked with animal and human diseases (Figure 1; Table S2). We also performed a *de novo* assembly with MetaSpades v.3.15.3 (Nurk et al., 2017) and obtained a total of 5,787 contigs (ranging from 125 - 6,752 nt, median size: 435 nt), with only two viral families identified (Asfarviridae with one contig classified by Kraken2 and Retroviridae with one contig classified by Diamond). Other viruses related to plants, phytoplankton and protozoa were also assigned, as Virgaviridae, Marnaviridae and Mimiviridae, respectively. Finally, a large amount of reads (Kraken2: n=576,822; Diamond: n=846,692) were not assigned, which potentially includes new and distantly related viral groups yet to be classified (Table S1). The available literature on sloths viral diseases mostly assesses their serological status, thus focusing on past exposure (Catenacci et al., 2018; Seymour, 1985). Our study shows the occurrence of current viral infections in sloth populations, also revealing the composition of their largely unknown fecal microbiome, as well as the presence of potentially pathogenic bacteria, and further contributes to the understanding of microorganism diversity. However, we emphasize the need for deeper sequencing to enable further analyses, such as phylogenetic studies and the intricate relationships between the bacteriome and its associated phages. We also highlight the importance of further studies on this topic, especially regarding its symbiont bacterial composition, which may be associated with the ability of this species to digest its food sources.

## Data Availability

Raw sequencing reads are available under BioProject accession number PRJNA971998 and NCBI SRA accession number SRR24525879. Assembled contigs, taxonomic assignment files and Krona plots are available on the project GitHub page (link: https://github.com/lddv-ufrj/sloth_virome).

## Supplementary material

The following online material is available for this article:

Table S1 - Overview data of filtered reads (n=945,386 corresponding to 45.77% of the total sequenced) and assembled contigs (n=5,787) taxonomically classified by Diamond and Kraken2.

Table S2 - Viral families classified by Kraken2 and Diamond.

Table S3 - Bacterial genera classified by Kraken2.
