# Supplementary material for: Fecal Virome of Southeastern Maned Sloth (Bradypus crinitus) (Pilosa: Bradypodidae)
Source: Genet Mol Biol. 2025 May 9;48(2):e20240183. doi: 10.1590/1678-4685-GMB-2024-0183 (PMC12063671; doi:10.1590/1678-4685-GMB-2024-0183)
Supplement: Table S1 - [file 1415-4757-GMB-48-02-e20240183-s1.pdf]

Genetics and Molecular Biology
https://doi.org/10.1590/1678-4685-GMB-2024-0183

Supplementary to "Fecal Virome of Southeastern Maned Sloth (*Bradypus crinitus*) (Pilosa: Bradypodidae)"

Table 1 - Overview data of filtered reads (n=945,386 corresponding to 45.77% of the total sequenced) and assembled contigs (n=5,787) taxonomically classified by Diamond and Kraken2.

		**Diamond**	**Kraken2**
**No hits**	**Reads (%)**	846,692 (89.7%)	576,822 (61%)
	**Contigs (%)**	3,392 (58.6%)	4,577 (79%)
	**(min - max)**	(125 -1,840 nt)	(125 - 4,162 nt)
**Mapped reads**		98,694 (10.4%)	368,564 (38.9%)
**Mapped contigs**		2,395	1,210
**(min - max)**		(186 - 6,752 nt)	(129 - 6,752 nt)
**Bacteria**	**Reads (%)**	54,910 (55.6%)	113,958 (30.9%)
	**Contigs (%)**	508 (21.2%)	511 (42.2%)
	**(min - max)**	(375 - 3,780 nt)	(131 - 4,802 nt)
**Eukaryota**	**Reads (%)**	42,975 (43.5%)	253,144 (68.7%)
	**Contigs (%)**	1,883 (78.6%)	683 (56.5%)
	**(min - max)**	(186 - 6,752 nt)	(129 - 6,752 nt)
**Viral**	**Reads (%)**	294 (0.3%)	318 (0.09%)
	**Contigs (%)**	2 (0.08%)	1 (0.08%)
	**(min - max)**	(381 - 1,441 nt)	(409 nt)
**Others/ Unassigned**	**Reads (%)**	515 (0.52%)	1,144 (0.31%)
	**Contigs (%)**	2 (0.08%)	15 (1.24%)
	**(min - max)**	(437 - 445 nt)	(186 - 735 nt)
