# Supplementary material for: Fecal Virome of Southeastern Maned Sloth (Bradypus crinitus) (Pilosa: Bradypodidae)
Source: Genet Mol Biol. 2025 May 9;48(2):e20240183. doi: 10.1590/1678-4685-GMB-2024-0183 (PMC12063671; doi:10.1590/1678-4685-GMB-2024-0183)
Supplement: Table S2 - [file 1415-4757-GMB-48-02-e20240183-s2.pdf]

Genetics and Molecular Biology
https://doi.org/10.1590/1678-4685-GMB-2024-0183

Supplementary to "Fecal Virome of Southeastern Maned Sloth (*Bradypus crinitus*) (Pilosa: Bradypodidae)"

Table S2 - Viral families classified by Kraken2 and Diamond.

VIRUS FAMILY	KNOWN HOSTS	READS K / D*	CONTIGS K / D*
**Adenoviridae****	**Vertebrates**	4 / 0	0 / 0
**Asfarviridae**	**Vertebrates** | Invertebrates	**2 / 0**	**1 / 0**
Autographiviridae	Bacteria	6 / 6	0 / 0
**Circoviridae**	**Vertebrates**	**0 / 8**	**0 / 0**
**CRESS viruses**	**Vertebrates** | Environmental	**0 / 3**	**0 / 0**
**Fimoviridae**	**Vertebrates**	**6 / 0**	**0 / 0**
**Herpesviridae**	**Vertebrates**	**10 / 0**	**0 / 0**
Marnaviridae	Phytoplankton	0 / 8	0 / 0
Microviridae	Bacteria	0 / 14	0 / 0
Mimiviridae	Protozoa	14 / 0	0 / 0
Myoviridae	Bacteria	34 / 46	0 / 0
**Papillomaviridae**	**Vertebrates**	**44 / 15**	**0 / 0**
**Parvoviridae**	**Vertebrates** | Invertebrates	**0 / 7**	**0 / 0**
Phycodnaviridae	Algae	8 / 1	0 / 0
Podoviridae	Bacteria	6 / 22	0 / 0
Polydnaviridae	Invertebrates	2 / 0	0 / 0
**Polyomaviridae**	**Vertebrates**	**6 / 0**	**0 / 0**
**Poxviridae**	**Vertebrates**	**4 / 0**	**0 / 0**
**Retroviridae**	**Vertebrates**	**2 / 23**	**0 / 1**
**Rhabdoviridae**	**Vertebrates** | Invertebrates | Plants	**0 / 2**	**0 / 0**
Salasmaviridae	Bacteria	2 / 3	0 / 0
Schitoviridae	Bacteria	4 / 8	0 / 0
Siphoviridae	Bacteria	96 / 72	0 / 0
Virgaviridae	Plants	2 / 0	0 / 0

*K = Kraken2 and D = Diamond.

** Featured data on viral families that infect vertebrates.
