# Supplementary material for: Fecal Virome of Southeastern Maned Sloth (Bradypus crinitus) (Pilosa: Bradypodidae)
Source: Genet Mol Biol. 2025 May 9;48(2):e20240183. doi: 10.1590/1678-4685-GMB-2024-0183 (PMC12063671; doi:10.1590/1678-4685-GMB-2024-0183)
Supplement: Table S3 - [file 1415-4757-GMB-48-02-e20240183-s3.pdf]

Genetics and Molecular Biology
https://doi.org/10.1590/1678-4685-GMB-2024-0183

Supplementary to "Fecal Virome of Southeastern Maned Sloth (*Bradypus crinitus*) (Pilosa: Bradypodidae)"

Table S3 - Bacterial genera classified by Kraken2.

BACTERIA GENUS	KRAKEN (Reads)
Corynebacterium	1620
Dietzia	32
Mycolicibacterium	214
Mycobacterium	132
Mycobacteroides	14
Mycolicibacter	12
Rhodococcus	100
Nocardia	72
Gordonia	56
Skermania	2
Tsukamurella	14
Tomitella	6
Lawsonella	36
Brevibacterium	106
Brachybacterium	36
Dermabacter	12
Rothia	500
Kocuria	136
Arthrobacter	58
Micrococcus	554
Pseudarthrobacter	24
Glutamicibacter	20
Citricoccus	2
Sinomonas	2
Paenarthrobacter	4
Zhihengliuella	4
Auritidibacter	8
Microbacterium	282
Curtobacterium	28
Agromyces	42
Agrococcus	2
Rathayibacter	16
Leucobacter	8
Leifsonia	18
Plantibacter	4
Salinibacterium	10
Clavibacter	6
Frondihabitans	6
Protaetiibacter	14
Aurantimicrobium	18
Humibacter	12
Microterricola	8
Aquiluna	14
Gryllotalpicola	4

BACTERIA GENUS	KRAKEN (Reads)
Agreia	6
Cellulomonas	30
Oerskovia	4
Actinotalea	2
Janibacter	138
Intrasporangium	12
Phycicoccus	14
Pedococcus	2
Serinicoccus	32
Ornithinimicrobium	26
Cellulosimicrobium	6
Isoptericola	2
Xylanimonas	6
Georgenia	14
Dermacoccus	68
Luteipulveratus	2
Beutenbergia	2
Ruania	6
Sanguibacter	4
Kytococcus	28
Actinomyces	266
Schaalia	806
Arcanobacterium	2
Pauljensenia	6
Mobiluncus	6
Actinobaculum	4
Trueperella	6
Gardnerella	122
Streptomyces	654
Streptacidiphilus	14
Kitasatospora	14
Arachnia	26
Cutibacterium	13692
Tessaracoccus	40
Propionibacterium	8
Microlunatus	56
Acidipropionibacterium	26
Auraticoccus	10
Raineyella	6
Brevilactibacter	24
Nocardioides	220
Aeromicrobium	10
Pimelobacter	4
Marmoricola	2
Mumia	2
Friedmanniella	22
Micropruina	8
Kribbella	8
Amycolatopsis	88
Pseudonocardia	48
Saccharomonospora	10
Kutzneria	14
Saccharopolyspora	20
Kibdelosporangium	2
Actinoalloteichus	14
Actinosynnema	8

BACTERIA GENUS	KRAKEN (Reads)
Saccharothrix	28
Lentzea	10
Alloactinosynnema	6
Micromonospora	110
Actinoplanes	22
Actinocatenispora	4
Plantactinospora	10
Catellatospora	4
Salinispora	4
Polymorphospora	2
Phytohabitans	6
Nonomuraea	12
Thermobispora	2
Actinomadura	8
Thermomonospora	8
Nocardiopsis	8
Streptomonospora	4
Frankia	14
Nakamurella	12
Stackebrandtia	2
Catenulispora	8
Jiangella	10
Blastococcus	20
Modestobacter	12
Epidermidibacterium	8
Kineococcus	2
Candidatus Planktophila	50
Arabia	2
Adlercreutzia	2
Aquihabitans	6
Ilumatobacter	2
Rubrobacter	894
Baekduia	8
Conexibacter	8
Euzebya	8
Egicoccus	12
Staphylococcus	1744
Jeotgalicoccus	28
Mammaliicoccus	34
Macrococcus	40
Bacillus	574
Lysinibacillus	52
Priestia	54
Virgibacillus	30
Cytobacillus	40
Alkalihalobacillus	24
Peribacillus	38
Psychrobacillus	12
Oceanobacillus	4
Metabacillus	22
Neobacillus	16
Halobacillus	10
Anoxybacillus	50
Heyndrickxia	20
Geobacillus	68
Fictibacillus	8

BACTERIA GENUS	KRAKEN (Reads)
Salicibibacter	8
Rossellomorea	6
Sutcliffiella	14
Pontibacillus	12
Anaerobacillus	8
Parageobacillus	2
Mesobacillus	2
Weizmannia	180
Lentibacillus	6
Radiobacillus	10
Caldibacillus	5468
Niallia	20
Paraliobacillus	6
Sediminibacillus	2
Pradoshia	8
Aeribacillus	30
Paenibacillus	1994
Brevibacillus	22
Cohnella	10
Aneurinibacillus	4
Saccharibacillus	8
Planococcus	58
Sporosarcina	14
Viridibacillus	4
Ureibacillus	8
Paenisporosarcina	2
Kurthia	4
Solibacillus	6
Rummeliibacillus	4
Gemella	88
Exiguobacterium	26
Listeria	42
Brochothrix	2
Alicyclobacillus	8
Tumebacillus	6
Streptococcus	2146
Enterococcus	78
Tetragenococcus	16
Vagococcus	20
Lactobacillus	130
Pediococcus	14
Lactiplantibacillus	26
Companilactobacillus	16
Ligilactobacillus	8
Limosilactobacillus	4
Latilactobacillus	18
Lacticaseibacillus	10
Loigolactobacillus	6
Bombilactobacillus	6
Liquorilactobacillus	4
Fructilactobacillus	2
Levilactobacillus	6
Paucilactobacillus	2
Aerococcus	12
Abiotrophia	482
Suicoccus	2

BACTERIA GENUS	KRAKEN (Reads)
Carnobacterium	46
Jeotgalibaca	10
Dolosigranulum	34
Clostridium	374
Alkaliphilus	24
Caloramator	12
Crassaminicella	8
Sarcina	4
Coprococcus	8
Lachnoanaerobaculum	10
Roseburia	36
Mediterraneibacter	24
Butyrivibrio	10
Anaerostipes	6
Anaerocolumna	18
Faecalicatena	10
Pseudobutyrivibrio	2
Anaerobutyricum	46
Cellulosilyticum	4
Herbinix	4
Faecalibacterium	18
Ruminococcus	12
Acetivibrio	6
Oscillibacter	8
Caproiciproducens	2
Ruminiclostridium	24
Monoglobus	2
Thermoclostridium	8
Pseudoclostridium	4
Caproicibacterium	4
Clostridioides	42
Romboutsia	12
Paraclostridium	4
Acetoanaerobium	2
Paeniclostridium	8
Desulfitobacterium	4
Desulfotomaculum	28
Mogibacterium	6
Aminipila	8
Thermaerobacter	2
Intestinimonas	6
Eubacterium	4
Alkalibacter	2
Candidatus Syntrophocurvum	2
Alkalicella	2
Caldicellulosiruptor	12
Thermoanaerobacterium	14
Caldanaerobacter	4
Moorella	6
Thermoanaerobacter	16
Thermodesulfobium	2
Halanaerobium	4
Natranaerobius	2
Selenomonas	20
Pectinatus	12
Pelosinus	6

BACTERIA GENUS	KRAKEN (Reads)
Methylomusa	2
Veillonella	1248
Dialister	4
Amedibacterium	4
Erysipelothrix	4
Turicibacter	6
Anaerococcus	16
Parvimonas	2
Helcococcus	2
Finegoldia	32
Peptoniphilus	4
Murdochiella	8
Schnuerera	2
Tissierella	22
Keratinibaculum	2
Nostoc	74
Anabaena	14
Cylindrospermum	4
Richelia	2
Calothrix	48
Anabaenopsis	10
Sphaerospermopsis	6
Dolichospermum	2
Scytonema	4
Brasilonema	8
Rivularia	14
Fischerella	12
Synechococcus	42
Cyanobium	2
Prochlorococcus	40
Acaryochloris	2
Leptolyngbya	42
Pseudanabaena	8
Chamaesiphon	48
Synechocystis	12
Rippkaea	2
Gloeothece	24
Euhalothece	6
Halothece	8
Candidatus Atelocyanobacterium	8
Geminocystis	36
Microcystis	32
Oxynema	2
Trichodesmium	8
Arthrospira	2
Microcoleus	8
Moorea	4
Oscillatoria	16
Cyanothece	2
Geitlerinema	2
Stanieria	2
Chroococcidiopsis	108
Mycoplasma	50
Mycoplasmopsis	32
Ureaplasma	10
Spiroplasma	58

BACTERIA GENUS	KRAKEN (Reads)
Mesoplasma	16
Acholeplasma	26
Candidatus Phytoplasma	2
Candidatus Izimaplasma	2
Deinococcus	100
Thermus	68
Ktedonosporobacter	4
Dehalococcoides	8
Fimbriimonas	8
Moraxella	2648
Acinetobacter	2664
Psychrobacter	44
Pseudomonas	5210
Entomomonas	10
Azotobacter	28
Oblitimonas	2
Aggregatibacter	44
Haemophilus	258
Pasteurella	14
Mannheimia	6
Rodentibacter	2
Actinobacillus	22
Glaesserella	4
Avibacterium	4
Histophilus	4
Klebsiella	184
Escherichia	168
Enterobacter	316
Citrobacter	64
Candidatus Blochmannia	6
Candidatus Gullanella	2
Salmonella	22
Leclercia	46
Kosakonia	12
Kluyvera	6
Raoultella	22
Jejubacter	2
Cronobacter	6
Cedecea	2
Yokenella	2
Buttiauxella	4
Phytobacter	4
Pseudescherichia	6
Proteus	62
Providencia	32
Morganella	8
Xenorhabdus	8
Photorhabdus	26
Arsenophonus	6
Pantoea	106
Buchnera	42
Erwinia	34
Tatumella	8
Mixta	14
Serratia	194
Yersinia	68

BACTERIA GENUS	KRAKEN (Reads)
Rahnella	18
Rouxiella	2
Candidatus Fukatsuia	6
Gibbsiella	4
Nissabacter	6
Pectobacterium	10
Dickeya	40
Edwardsiella	26
Hafnia	6
Pragia	4
Sodalis	10
Plesiomonas	4
Pseudoalteromonas	126
Psychrosphaera	2
Alteromonas	40
Marinobacter	20
Paraglaciecola	2
Catenovulum	4
Glaciecola	10
Shewanella	120
Parashewanella	2
Colwellia	24
Thalassotalea	10
Litorilituus	2
Idiomarina	2
Moritella	14
Psychromonas	2
Vibrio	226
Photobacterium	10
Salinivibrio	6
Enterovibrio	4
Aliivibrio	10
Paraphotobacterium	2
Halomonas	142
Candidatus Carsonella	2
Candidatus Portiera	2
Kushneria	10
Pistricoccus	6
Marinomonas	26
Marinobacterium	4
Bacterioplanes	4
Alcanivorax	58
Spartinivicinus	2
Hahella	2
Reinekea	2
Gynuella	6
Kangiella	6
Stenotrophomonas	270
Xanthomonas	174
Lysobacter	222
Luteimonas	66
Thermomonas	140
Arenimonas	16
Xylella	4
Pseudoxanthomonas	70
Aerosticca	14

BACTERIA GENUS	KRAKEN (Reads)
Rhodanobacter	138
Dyella	168
Luteibacter	24
Cardiobacterium	18
Francisella	36
Allofrancisella	2
Piscirickettsia	8
Thiomicrorhabdus	14
Cycloclasticus	2
Thiosulfatimonas	4
Beggiatoa	4
Thiothrix	8
Legionella	88
Tatlockia	4
Coxiella	2
Aquicella	4
Cellvibrio	18
Simiduia	2
Teredinibacter	2
Microbulbifer	6
Congregibacter	4
Halioglobus	4
Zhongshania	2
Rheinheimera	12
Allochromatium	4
Nitrosococcus	4
Alkalilimnicola	4
Acidihalobacter	6
Ectothiorhodospira	4
Spiribacter	8
Sulfurivermis	12
Halothiobacillus	6
Aeromonas	314
Oceanimonas	2
Zobellella	4
Succinivibrio	2
Candidatus Vesicomyosocius	24
Candidatus Thioglobus	2
Candidatus Reidiella	2
Candidatus Pseudothioglobus	8
Methylomonas	14
Methylococcus	28
Methylovulum	2
Frischella	8
Gilliamella	2
Sinimarinibacterium	4
Neisseria	812
Kingella	20
Eikenella	8
Simonsiella	6
Chromobacterium	22
Iodobacter	2
Aquaspirillum	6
Vogesella	8
Chitinibacter	6
Paludibacterium	10

BACTERIA GENUS	KRAKEN (Reads)
Aquitalea	202
Jeongeupia	14
Microvirgula	4
Lautropia	54
Burkholderia	4626
Cupriavidus	406
Polynucleobacter	866
Paraburkholderia	1506
Pandoraea	106
Ralstonia	11656
Chitinimonas	6
Mycoavidus	4
Caballeronia	50
Acidovorax	1612
Ottowia	54
Comamonas	774
Variovorax	260
Hydrogenophaga	160
Delftia	1072
Rhodoferax	120
Diaphorobacter	72
Limnohabitans	46
Verminephrobacter	18
Polaromonas	36
Ramlibacter	32
Melaminivora	18
Hylemonella	28
Alicycliphilus	54
Achromobacter	140
Bordetella	114
Alcaligenes	14
Pelistega	2
Pigmentiphaga	10
Paenalcaligenes	2
Kerstersia	4
Pusillimonas	4
Castellaniella	42
Orrella	2
Algicoccus	2
Massilia	188
Herbaspirillum	84
Janthinobacterium	74
Collimonas	48
Duganella	46
Herminiimonas	4
Noviherbaspirillum	4
Rubrivivax	34
Thiomonas	20
Rhizobacter	82
Xylophilus	12
Roseateles	34
Paucibacter	18
Sutterella	12
Candidatus Methylopumilus	26
Methylophilus	4
Methylovorus	8

BACTERIA GENUS	KRAKEN (Reads)
Nitrosomonas	22
Nitrosospira	6
Denitratisoma	20
Sulfuriferula	8
Sulfuritortus	38
Azospira	18
Aromatoleum	24
Rugosibacter	4
Azoarcus	42
Thauera	42
Dechloromonas	22
Candidatus Kinetoplastibacterium	2
Casimicrobium	22
Rhizobium	572
Agrobacterium	112
Neorhizobium	38
Sinorhizobium	70
Ensifer	34
Liberibacter	4
Shinella	16
Bradyrhizobium	820
Rhodopseudomonas	52
Tardiphaga	18
Nitrobacter	20
Mesorhizobium	410
Nitratireductor	14
Phyllobacterium	28
Aminobacter	24
Hoeflea	34
Lentilitoribacter	4
Methylobacterium	1378
Microvirga	56
Methylorubrum	498
Bartonella	56
Labrenzia	58
Pseudovibrio	2
Stappia	12
Pannonibacter	10
Ancylobacter	14
Labrys	10
Pseudolabrys	10
Xanthobacter	16
Starkeya	18
Devosia	68
Brucella	44
Hyphomicrobium	16
Methyloceanibacter	6
Rhodoplanes	6
Martelella	38
Aureimonas	50
Bosea	120
Candidatus Phaeomarinobacter	6
Kaustia	2
Phreatobacter	320
Methylocella	6
Methylocystis	4

BACTERIA GENUS	KRAKEN (Reads)
Kaistia	8
Hartmannibacter	6
Chelatococcus	40
Pseudorhodoplanes	6
Sphingomonas	2776
Sphingobium	632
Sphingopyxis	216
Novosphingobium	142
Sphingosinithalassobacter	84
Parasphingopyxis	4
Sphingorhabdus	32
Rhizorhabdus	8
Tardibacter	10
Novosphingopyxis	8
Erythrobacter	34
Altererythrobacter	28
Qipengyuania	16
Paraurantiacibacter	8
Tsuneonella	18
Sphingosinicella	30
Paracoccus	246
Cereibacter	14
Rhodovulum	18
Pseudorhodobacter	2
Thioclava	2
Rhodobacter	32
Pacificitalea	2
Pukyongiella	6
Qingshengfaniella	2
Profundibacter	2
Pseudopuniceibacterium	4
Pontivivens	8
Gemmobacter	4
Haematobacter	14
Sulfitobacter	38
Salipiger	18
Sagittula	4
Phaeobacter	10
Dinoroseobacter	2
Ketogulonicigenium	6
Ruegeria	12
Maribius	2
Marinovum	10
Roseovarius	16
Acetobacter	6
Roseomonas	82
Komagataeibacter	20
Gluconobacter	12
Acidibrevibacterium	2
Granulibacter	10
Roseococcus	2
Lichenicola	18
Azospirillum	60
Skermanella	22
Nitrospirillum	8
Magnetospirillum	18

BACTERIA GENUS	KRAKEN (Reads)
Tistrella	20
Defluviicoccus	4
Candidatus Endolissoclinum	4
Indioceanicola	8
Thalassospira	8
Magnetospira	4
Elioraea	14
Stella	14
Wolbachia	16
Ehrlichia	18
Anaplasma	2
Rickettsia	36
Caulobacter	134
Brevundimonas	2836
Phenylobacterium	12
Candidatus Cytomitobacter	4
Candidatus Pelagibacter	24
Magnetococcus	4
Marinicauda	8
Campylobacter	80
Aliarcobacter	20
Arcobacter	40
Malaciobacter	30
Pseudoarcobacter	2
Halarcobacter	8
Sulfurospirillum	32
Helicobacter	30
Sulfurimonas	28
Nautilia	6
Nitratiruptor	4
Archangium	20
Cystobacter	2
Myxococcus	22
Corallococcus	14
Pyxidicoccus	6
Vulgatibacter	2
Anaeromyxobacter	12
Sorangium	30
Chondromyces	4
Labilithrix	2
Desulfovibrio	30
Pseudodesulfovibrio	4
Lawsonia	8
Maridesulfovibrio	8
Geobacter	40
Geomonas	6
Desulfonema	12
Desulfobacter	2
Desulfobacterium	4
Desulfosarcina	4
Desulfobacula	10
Desulfotalea	4
Syntrophobacter	4
CandidatusDesulfofervidus	2
Bradymonas	4
Persicimonas	4

BACTERIA GENUS	KRAKEN (Reads)
Hippea	2
Halobacteriovorax	2
Bdellovibrio	12
Silvanigrella	4
Acidithiobacillus	22
Prevotella	1406
Alloprevotella	6
Porphyromonas	22
Tannerella	42
Alistipes	16
Dysgonomonas	6
Petrimonas	6
Butyricimonas	12
Odoribacter	2
Tenuifilum	8
Salinivirga	8
Coprobacter	4
Maribellus	6
Sphingobacterium	166
Pedobacter	94
Mucilaginibacter	112
Pseudopedobacter	6
Olivibacter	4
Anseongella	2
Capnocytophaga	250
Flavobacterium	566
Polaribacter	94
Maribacter	24
Winogradskyella	32
Tenacibaculum	54
Formosa	34
Nonlabens	30
Aquimarina	16
Myroides	24
Cellulophaga	50
Gramella	16
Kordia	10
Lacinutrix	14
Olleya	16
Algibacter	8
Seonamhaeicola	10
Lutibacter	6
Dokdonia	10
Urechidicola	14
Muricauda	8
Flavivirga	6
Salegentibacter	26
Galbibacter	2
Amniculibacterium	10
Mesoflavibacter	8
Flagellimonas	4
Psychroserpens	6
Wenyingzhuangia	10
Altibacter	2
Pukyongia	2
Chryseobacterium	312

BACTERIA GENUS	KRAKEN (Reads)
Kaistella	44
Epilithonimonas	70
Elizabethkingia	38
Bergeyella	6
Empedobacter	364
Cloacibacterium	120
Riemerella	2
Ornithobacterium	4
Weeksella	4
Blattabacterium	42
Fluviicola	4
Owenweeksia	8
Rufibacter	6
Hymenobacter	36
Pontibacter	12
Adhaeribacter	2
Spirosoma	50
Runella	10
Fibrella	2
Cytophaga	8
Arcticibacterium	12
Echinicola	2
Algoriphagus	28
Cyclobacterium	4
Aquiflexum	6
Belliella	2
Flammeovirga	22
Candidatus Amoebophilus	4
Bernardetia	14
Chitinophaga	16
Arachidicoccus	16
Phnomibacter	2
Filimonas	6
Pseudobacter	16
Niastella	14
Flavisolibacter	2
Panacibacter	26
Niabella	8
Paraflavitalea	8
Saprospira	2
Haliscomenobacter	2
Melioribacter	4
Gemmatirosa	10
Treponema	52
Borrelia	18
Borreliella	18
Thiospirochaeta	4
Leptospira	30
Brachyspira	12
Fusobacterium	158
Leptotrichia	100
Pseudoleptotrichia	2
Streptobacillus	8
Sebaldella	8
Gimesia	18
Planctomyces	4

BACTERIA GENUS	KRAKEN (Reads)
Rubinisphaera	4
Crateriforma	2
Maioricimonas	6
Symmachiella	2
Thalassoglobus	10
Lignipirellula	4
Mariniblastus	2
Bremerella	2
Stieleria	8
Pirellulimonas	10
Gemmata	16
Singulisphaera	4
Aquisphaera	4
Paludisphaera	2
Sedimentisphaera	2
Limihaloglobus	2
Candidatus Kuenenia	6
Chlamydia	14
Candidatus Protochlamydia	8
Opitutus	4
Verrucomicrobium	6
Akkermansia	8
Kiritimatiella	4
Fervidobacterium	6
Thermosipho	16
Pseudothermotoga	2
Mesotoga	4
Kosmotoga	4
Defluviitoga	2
Terriglobus	12
Acidobacterium	2
Granulicella	8
Candidatus Solibacter	6
Chloracidobacterium	2
Sulfurihydrogenibium	10
Persephonella	4
Thermocrinis	6
Aquifex	2
Candidatus Nanosynbacter	10
Candidatus Babela	6
Endomicrobium	2
Nitrospira	6
Calditerrivibrio	8
Atribacter	2
Caldithrix	4
Haloarcula	8
Halosimplex	8
Halorhabdus	6
Candidatus Halobonum	2
Halorubrum	4
Haloferax	2
Haloplanus	4
Natrialba	12
Haloterrigena	8
Natronorubrum	2
Methanosarcina	50

BACTERIA GENUS	KRAKEN (Reads)
Methanohalophilus	4
Methanococcoides	8
Methanothrix	2
Methanoculleus	2
Methanospirillum	8
Thermococcus	10
Pyrococcus	2
Methanobacterium	6
Methanobrevibacter	8
Methanosphaera	24
Methanothermus	4
Methanocaldococcus	16
Methanotorris	2
Methanococcus	12
Acidianus	10
Sulfolobus	12
Sulfurisphaera	2
Saccharolobus	4
Aeropyrum	2
Nitrosopumilus	48
Nitrosarchaeum	2
Candidatus Nitrosocosmicus	2
Candidatus Nitrosopelagicus	4
Thermoplasma	8
Aciduliprofundum	2
Candidatus Prometheoarchaeum	8
Simplicispira	10
Kinneretia	50
Curvibacter	16
Undibacterium	96
Advenella	6
Turicimonas	8
Vitreoscilla	14
Laribacter	6
Methylotenera	28
Usitatibacter	2
Niveibacterium	10
Thioalkalivibrio	18
Thiodictyon	2
Cobetia	4
Salinicola	4
Fluoribacter	2
Rickettsiella	2
Methylocaldum	4
Orbus	4
Salinisphaera	6
Oricola	4
Roseitalea	16
Afipia	4
Aquabacter	20
Azorhizobium	14
Parvibaculum	4
Lichenihabitans	2
Aurantiacibacter	2
Zymomonas	12
Gluconacetobacter	8

BACTERIA GENUS	KRAKEN (Reads)
Niveispirillum	10
Hypericibacter	16
Rhodospirillum	4
Epibacterium	2
Mameliella	4
Candidatus Paracaedibacter	8
Hyphobacterium	2
Poseidonibacter	6
Nitrosophilus	2
Desulfopila	4
Mariprofundus	4
Hydrogenophilus	580
Salimicrobium	4
Salinicoccus	2
Abyssicoccus	4
Thermobacillus	6
Apilactobacillus	2
Flintibacter	4
Halothermothrix	2
Koleobacter	2
Luteimicrobium	2
Flaviflexus	2
Scardovia	2
Candidatus Nanopelagicus	4
Actinomarinicola	8
Chondrocystis	4
Changchengzhania	6
Zunongwangia	2
Mariniflexile	8
Psychroflexus	2
Planobacterium	34
Candidatus Sulcia	6
Draconibacterium	8
Candidatus Cardinium	784
Marivirga	18
Chryseolinea	8
Lacibacter	14
Pseudocnuella	6
Gemmatimonas	8
Sneathia	2
Calycomorphotria	2
Tuwongella	4
Limnoglobus	2
Urbifossiella	8
Parachlamydia	4
Simkania	4
Ereboglobus	2
Marinitoga	4
Methanoregula	4
Halorussus	4
Archaeoglobus	2
Candidatus Nitrosotenuis	20
Stygiolobus	2
Schlegelella	134
Methylibium	50
Ferrigenium	4

BACTERIA GENUS	KRAKEN (Reads)
Fluviibacter	24
Candidatus Profftella	2
Shigella	4
Methylophaga	8
Sedimenticola	2
Pseudohongiella	2
Nordella	2
Candidatus Hodgkinia	10
Blastochloris	4
Porphyrobacter	30
Pseudohalocynthiibacter	6
Pelagovum	6
Neokomagataea	2
Micavibrio	6
Hyphomonas	8
Rhodoluna	8
Arsenicicoccus	6
Fannyhessea	2
Sporolactobacillus	6
Novibacillus	4
Thermosynechococcus	6
Thermogutta	4
Tautonia	6
Ruthenibacterium	10
Heliorestis	2
Sedimentibacter	4
Renibacterium	2
Tropheryma	2
Koinonema	2
Halotalea	2
Thalassolituus	4
Caldichromatium	2
Woeseia	8
Guyparkeria	2
Gallaecimonas	2
Solimonas	4
Chitinolyticbacter	6
Pseudogulbenkiania	8
Mitsuaria	28
Sulfurimicrobium	4
Oryzomicrobium	4
Sandaracinus	8
Desulfurivibrio	2
Desulfocapsa	4
Desulfolutivibrio	4
Beijerinckia	10
Croceicoccus	34
Commensalibacter	2
Pelagibaca	4
Octadecabacter	4
Pikeienuella	8
Tamlana	4
Nibribacter	18
Dyadobacter	16
Fulvivirga	6
Ignavibacterium	6

BACTERIA GENUS	KRAKEN (Reads)
Sediminispirochaeta	2
Roseimicrobium	4
Lacunisphaera	2
Candidatus Saccharimonas	8
Halolamina	2
Agarivorans	2
Ferrimonas	6
Kineobactrum	2
Thiocystis	2
Marichromatium	6
Ciceribacter	2
Variibacter	6
Pyruvatibacter	12
Citromicrobium	12
Sandaracinobacter	10
Ferrovibrio	2
Pararhodospirillum	2
Roseivivax	4
Actibacterium	4
Celeribacter	12
Parasedimentitalea	4
Paraoceanicella	10
Hirschia	6
Pulveribacter	14
Serpentinomonas	30
Leptothrix	36
Aquabacterium	32
Sulfuritalea	6
Gloeocapsa	4
Oceanithermus	6
Truepera	4
Dehalogenimonas	2
Euzebyella	4
Rhodopirellula	6
Roseimaritima	2
Lacipirellula	4
Frigoriglobus	4
Hydrogenobaculum	8
Deferribacter	6
Herbiconiux	4
Microcella	2
Tetrasphaera	6
Yimella	2
Geodermatophilus	72
Lancefieldella	2
Slackia	4
Kroppenstedtia	8
Laceyella	2
Fastidiosipila	4
Peptoclostridium	2
Ammonifex	2
Cylindrospermopsis	4
Roseiflexus	4
Tepidiforma	10
Rhodothermus	6
Scandinavium	14

BACTERIA GENUS	KRAKEN (Reads)
Frateuria	12
Saccharospirillum	4
Thioflavicoccus	2
Thermochromatium	6
Sulfurifustis	2
Sulfuricaulis	4
Pseudorhizobium	2
Peteryoungia	4
Chelativorans	10
Filomicrobium	2
Methyloligella	2
Caenibius	2
Paradevosia	4
Maritalea	2
Youhaiella	8
Breoghania	12
Tabrizicola	6
Defluviimonas	16
Pseudooceanicola	2
Roseicitreum	4
Antarctobacter	2
Roseibacterium	6
Allosphingosinicella	12
Blastomonas	46
Pelagerythrobacter	6
Acidiphilium	10
Neoasaia	2
Terasakiella	2
Asticcacaulis	4
Terricaulis	26
Candidatus Symbiobacter	4
Inhella	12
Crenobacter	10
Deefgea	2
Methyloversatilis	6
Sulfuricella	4
Gallionella	4
Thiobacillus	4
Candidatus Accumulibacter	6
Melittangium	2
Desulfococcus	4
Desulfarculus	2
Aeoliella	2
Alienimonas	2
Methylacidimicrobium	2
Waddlia	2
Thermovibrio	2
Natrinema	10
Halovivax	4
Desulfurococcus	2
Methylovirgula	10
Muriicola	6
Ilyobacter	2
Candidatus Vallotia	2
